# Anatomical topology of extrahippocampal projections from dorsoventral CA pyramidal neurons in mice

**DOI:** 10.3389/fnana.2024.1421034

**Published:** 2024-07-23

**Authors:** Junseop Lee, Jeongrak Park, Minseok Jeong, Seo-Jin Oh, Jong-Hyuk Yoon, Yong-Seok Oh

**Affiliations:** ^1^Molecular Psychiatry Laboratory, Brain Sciences, Daegu Gyeongbuk Institute of Science and Technology, Daegu, Republic of Korea; ^2^Neurodegenerative Diseases Research Group, Korea Brain Research Institute, Daegu, Republic of Korea

**Keywords:** hippocampus, extrahippocampal projection, lateral septum, CA pyramidal neurons, dorsoventral axis

## Abstract

The hippocampus primarily functions through a canonical trisynaptic circuit, comprised of dentate granule cells and CA1-CA3 pyramidal neurons (PNs), which exhibit significant heterogeneity along the dorsoventral axis. Among these, CA PNs are known to project beyond the hippocampus into various limbic areas, critically influencing cognitive and affective behaviors. Despite accumulating evidence of these extrahippocampal projections, the specific topological patterns—particularly variations among CA PN types and between their dorsal and ventral subpopulations within each type—remain to be fully elucidated. In this study, we utilized cell type-specific Cre mice injected with fluorescent protein-expressing AAVs to label each CA PN type distinctly. This method further enabled the dual-fluorescence labeling of dorsal and ventral subpopulations using EGFP and tdTomato, respectively, allowing a comprehensive comparison of their axonal projections in an animal. Our findings demonstrate that CA1 PNs predominantly form unilateral projections to the frontal cortex (PFC), amygdala (Amy), nucleus accumbens (NAc), and lateral septum (LS), unlike CA2 and CA3 PNs making bilateral innervation to the LS only. Moreover, the innervation patterns especially within LS subfields differ according to the CA PN type and their location along the dorsoventral axis of the hippocampus. This detailed topographical mapping provides the neuroanatomical basis of the underlying functional distinctions among CA PN types.

## 1 Introduction

The hippocampus plays a pivotal role in memory, cognitive functions, and emotional regulation, as evidenced by foundational research (O’Keefe and Dostrovsky, 1971; Morris et al., 1982). It consists of 4 major subregions: the dentate gyrus (DG), CA3, CA2, and CA1. These regions receive afferent inputs from the superficial layers of the entorhinal cortex (EC) and primarily send efferent outputs back to the deeper layer of the EC. Within the trisynaptic circuit, the DG relays the initial inputs from the EC firstly to CA3 and these excitatory signals are transmitted sequentially to other CA subfields for the contextual processing of learning and memory. Furthermore, each subfield of the hippocampus comprises of excitatory principal neurons such as DG granule cells (GCs) and CA PNs, and various types of GABAergic interneurons.

Previous studies have demonstrated that each subregion is associated with distinctive functions ranging from aggression to social recognition (Hitti and Siegelbaum, 2014). Within the hippocampus, CA subregions not only form canonical trisynaptic circuits but also establish direct extrahippocampal connections with various limbic regions (Kim and Fanselow, 1992; Wu et al., 2018). The CA1 extends projections to various limbic areas such as the PFC, NAc, and Amy, which are critical centers for reward, decision-making, and emotional regulation (Dolorfo and Amaral, 1998; Cenquizca and Swanson, 2007; Hoover and Vertes, 2007; Schultz, 2010; Preston et al., 2013; Root et al., 2015). Notably, CA2 is primarily associated with social behavior, as previous studies have unveiled its involvement in social recognition memory through CA2-LS connections (Hitti and Siegelbaum, 2014; Pagani et al., 2015; Alexander et al., 2016). Meanwhile, previous research has shown that signaling from CA3 to the LS influences spatial memory and context discrimination (Smith and Mizumori, 2006; Hamilton et al., 2018). These extrahippocampal projections from each CA subarea are differentially associated with cognitive and affective functions.

The classical neuronal tracing approach has demonstrated that CA subregions of the hippocampus make direct extrahippocampal projections to various limbic regions (Bienkowski et al., 2018). Traditional methods using fluorescein-linked phaseolus vulgaris-leucoagglutinin (PHAL), an anterograde tracing dye, have been instrumental in the initial identification of the extrahippocampal connectivity of each hippocampal subfield (Amaral and Witter, 1989). These approaches have also been pivotal in mapping projections to various limbic areas, including the prefrontal cortex (PFC), amygdaloid complex (Amy), and nucleus accumbens (NAc) (Gaykema et al., 1991; Cenquizca and Swanson, 2007; Bienkowski et al., 2018). These studies underscore the extensive and diverse connectivity of hippocampal subregions with other limbic areas, highlighting the complexity of hippocampal function. However, these classical approaches have limitations, particularly in targeting specific neuronal types within hippocampal subfields. They are prone to non-selective labeling of multiple cell types, which can lead to misinterpretations of cell type-specific projection patterns. To address this issue, cell type-specific Cre drivers have been developed and widely used in numerous studies to delineate the function of each principal neuron (PN) type within the hippocampal neural circuit (Tsien et al., 1996; Nakazawa et al., 2002; Hitti and Siegelbaum, 2014). Despite these advancements, the extrahippocampal projection patterns of specific CA PN types, especially the Cre+ subpopulation, remain unclear, and a direct comparison among the three CA PN types has yet to be conducted.

The hippocampus is a long-curved structure that extends dorsoventrally from the septal nuclei of the forebrain to the temporal cortex (Strange et al., 2014). Furthermore, studies have documented molecular, cellular, and functional heterogeneity within the longitudinal structure of the hippocampus (McGowan-Sass, 1973; Moser and Moser, 1998; Bienkowski et al., 2018). For instance, rodent studies have shown that the dorsal region of the hippocampus is primarily associated with cognitive functions for spatial navigation and contextual processing. In contrast, the ventral region is more involved in the affective responses, including the regulation of emotions and anxiety (Fanselow and Dong, 2010; Poppenk et al., 2013). Thus, the hippocampus exhibits a significant functional heterogeneity along the dorsoventral axis. Despite functional differentiation within each Cre+ CA PNs along their dorsoventral axis, the topological pattern of their extrahippocampal connectivity has not been directly compared.

In this study, we constructed a comprehensive map of the extrahippocampal projections of each CA PN type, further analyzing these projections based on their dorsoventral position in the hippocampus. Utilizing targeted Cre driver lines for each Cre+ subpopulation of CA PNs, we visualized their dorsal or ventral pathways to limbic regions, revealing organized patterns of extrahippocampal connectivity. Our findings demonstrate a distinct unilateral projection pattern of CA1 PNs to critical limbic areas, in contrast to the bilateral connectivity exhibited by Cre+ populations of CA2 and CA3 PNs, which project only to the lateral septum (LS). Moreover, we illuminated three-dimensional variations in hippocampal-septal projection patterns based on the Cre+ subpopulation of CA PNs and its dorsoventral location within the hippocampus. Thus, this topographical mapping provides an anatomical framework for the functional differentiation among CA PN types via their direct connections to limbic areas.

## 2 Materials and methods

### 2.1 Animals

The mice used for the hippocampus-limbic connectivity analysis include both female and male mice (12–18 weeks old) that express Cre transgene specifically in each CA-PN type. These transgenic mouse lines include Camk2a-Cre mice (JAX#005359; Tsien et al., 1996), Amigo2-Cre (JAX#030215; Hitti and Siegelbaum, 2014), and Grik4-Cre (JAX#006474; Nakazawa et al., 2002) that are used to target CA1-, CA2-, and CA3-PNs, respectively. Mice were housed under a 12-h light-dark cycle in a specific pathogen-free (SPF) facility, with *ad libitum* access to food and water. All procedures were approved by the Animal Care and Use Committee of the DGIST (IACUC#, #20011503-03).

### 2.2 Stereotaxic surgery and virus injections

An AAV1-CAG-FLEX-eGFP-WPRE-bGH vector (UPenn vector core, originally developed by the Allen Institute) was employed to label PNs in the dorsal hippocampus region and to express enhanced green fluorescent protein (EGFP). Additionally, an AAV1-CAG-FLEX-tdTomato vector (UNC vector core; donated by Dr. Brian Roth) was used to label the connectivity from the ventral hippocampus to the LS with tdTomato fluorescence. Both viruses contain the FLEX sequence for Cre recombination, ensuring specificity in the fluorescence labeling in the target cell type. Both viruses contain the FLEX sequence for Cre recombination, ensuring specificity in the fluorescence labeling in the target cell type. Stereotaxic injections of AAV constructs (Both AAV stocks were diluted in 1.3 × 10^12^ GC/mL with 5% sorbitol in 1õ PBS) were precisely administered using an Angle Two™ stereotaxic frame designed for mice (Leica, Buffalo Grove, IL, USA). Prior to the stereotaxic injection of AAV constructs, mice were anesthetized with an intraperitoneal injection of Avertin at a dosage of 250 mg/kg. Cre-dependent AAVs were unilaterally injected 500 nl into dorsal and ventral regions of the CA1-CA3 (coordinates for dorsal CA1 (dCA1) = AP: −1.94 mm, ML: ± 1.40 mm, DV: −1.15 mm, dorsal CA2 (dCA2) = AP: −2.00 mm, ML: ± 2.40 mm, DV: −1.50 mm, dorsal CA3 (dCA3) = AP: −1.94 mm, ML: ± 2.10 mm, and DV: −2.00 mm, ventral CA1 (vCA1) = AP −3.30 mm, ML ± 2.70 mm, DV −3.60 mm, ventral CA2 (vCA2) = AP −3.30 mm, ML ± 2.70 mm, DV −3.60 mm, ventral CA3 (vCA3) = AP: −3.08 mm, ML: ± 2.50 mm, DV: −3.50 mm) using 10 μl Hamilton syringes (33 gauge needle; Reno, NV, USA). The flow rate of AAV injection (0.2 μl/min) was controlled with a nanopump controller (WPI, US). To minimize unnecessary diffusion along the needle track, the needle was left in place for 5 min post-injection, and the incision was then closed with a wound clip. Mice were placed back in their home cage to recover, and the wound clip was removed a week later, once the incision had completely healed. All animals were allowed at least 2 weeks of rest before proceeding to the next experimental stage. Any mice exhibiting abnormal recovery after stereotaxic surgery were sacrificed and excluded from further analysis.

### 2.3 Perfusion

Under deep anesthesia induced by Avertin, mice were perfused with phosphate-buffered saline (PBS), followed by 4% paraformaldehyde (PFA) in PBS. Brains were extracted, post-fixed in 4% PFA for 12 h, and subsequently stored in 30% sucrose at 4°C until sectioning. Brains were embedded in an OCT compound, sectioned at 40 μm using a cryostat (Leica VT1000s, Germany). Coronal sections were prepared from all brains. The entire brain was sectioned, and every second slice was mounted on glass slides, then sealed with coverslips using Prolong Gold antifade mountant (Thermo Fisher Scientific). Mounted slides were stored at 4°C until ready for imaging.

### 2.4 Fluorescence imaging

Injection site images were captured using a widefield Zeiss AxioObserver.Z1 microscope (Zeiss, Germany) equipped with DAPI, EGFP, and tdTomato filter cubes. These were excited by LEDs at wavelengths of 353 nm, 488 nm, and 555nm, respectively. For co-localization analysis, images were acquired with a high-resolution Carl Zeiss LSM 800 confocal microscope. We used objectives of 10õ with a 0.4 numerical aperture (NA) or 20õ with a 1.0 NA, employing a 405 nm laser for excitation. Additionally, a white light laser was set at 465 nm, 509 nm, and 581 nm for DAPI, EGFP, and tdTomato fluorescence, respectively, with optical emission filtering according to the defaults for each fluorophore. The images were captured at a resolution of 1024 × 1024 pixels with an accumulation setting of 2õ and bidirectional scanning. The pinhole was adjusted to 1 airy unit to optimize spatial resolution, and we collected a z-stack of four images across a 12 μm volume to capture sufficient depth. Following image acquisition, we used FUJI software for processing, specifically converting the images to maximum intensity z-projections for detailed analysis. This approach proved effective as the AAV-dependent labeling of PNs in the hippocampus and the fiber density of the LS displayed spatial sparsity within the z-imaging plane, allowing for clear delineation and analysis using maximum intensity projections. Laser intensity and detector settings were consistently maintained for each brain.

### 2.5 Fluorescence density analysis in the LS subfield

To map the axonal fiber distribution of Cre+ CA PNs, we conducted a detailed imaging analysis of serial coronal sections. Each brain slice was manually examined to identify the locations of fibers in the LS, using EGFP and tdTomato markers for visualization. We specifically marked each fiber based on its location within specific anatomical brain regions or subregions, facilitating targeted quantification. We divided the LS into four segments for a detailed quantitative analysis of axonal fibers along the rostrocaudal axis. According to the reference regarding the coronal sections of mouse brain (Allen Brain Atlas), we divided the region from Bregma +1.0 to +0.04 mm into intervals of 0.24 mm, designated as segments #1 to #4. Each segment consists of six brain sections, each with a thickness of 0.04 mm. To determine the density of each fiber, we measured the labeled axonal pixel area and divided it by the total LS area, excluding the MS in the septal region. These indexes were then normalized using the density of segment #4 (Bregma 0.28∼0.04 mm) as a baseline, given its proximity to the hippocampus. Furthermore, to analyze axonal fibers along the dorsoventral axis of the LS, we examined the regions from DV −2.50 to −3.50 mm (dorsal and intermediate LS), corresponding to segment #4. We defined ten segments at 0.1 mm intervals to assess the pixel density relative to the LS area along the dorsoventral axis. The MS was excluded in this analysis. We measured the fiber density by dividing the pixel index by the total segment area, and these indexes were normalized using the density of the segment with the highest value. All area and pixel measurements were carried out using ImageJ (NIH).

### 2.6 Cell counting in the hippocampus

Sections were collected from the representative dorsal and ventral hippocampus using the Allen brain atlas. The hippocampal subregions were identified by well-defined anatomical landmarks visualized with straining DAPI. The quantified CA region was defined as the fluorescence-labeled area on the pyramidal cell layer in the nearby injection sites. The double-labeling of viral fluorescence was defined by which EGFP or tdTomato was overlapped by the blue fluorescence of DAPI. The co-labeling portions were calculated by dividing the number of cells that were labeled with both markers by the total number of cells labeled with DAPI in the nearby injection sites. Labeled Cre+ CA PNs in each subregion were manually counted using ImageJ software.

### 2.7 Statistics

A comparison of two samples was analyzed by a parametric (two-tailed student *t*-test) test or nonparametric (Mann–Whitney test) test. Statistical analyses were performed using Prism 7.0a (Graphpad, La Jolla, CA). Statistical parameters and analysis performed can be found in the figure legends. All data are presented as mean ± standard error of the mean (SEM).

## 3 Result

### 3.1 Dual-colored, cell type-specific labeling of dorsal and ventral CA PNs in the hippocampus

**FIGURE 1:**
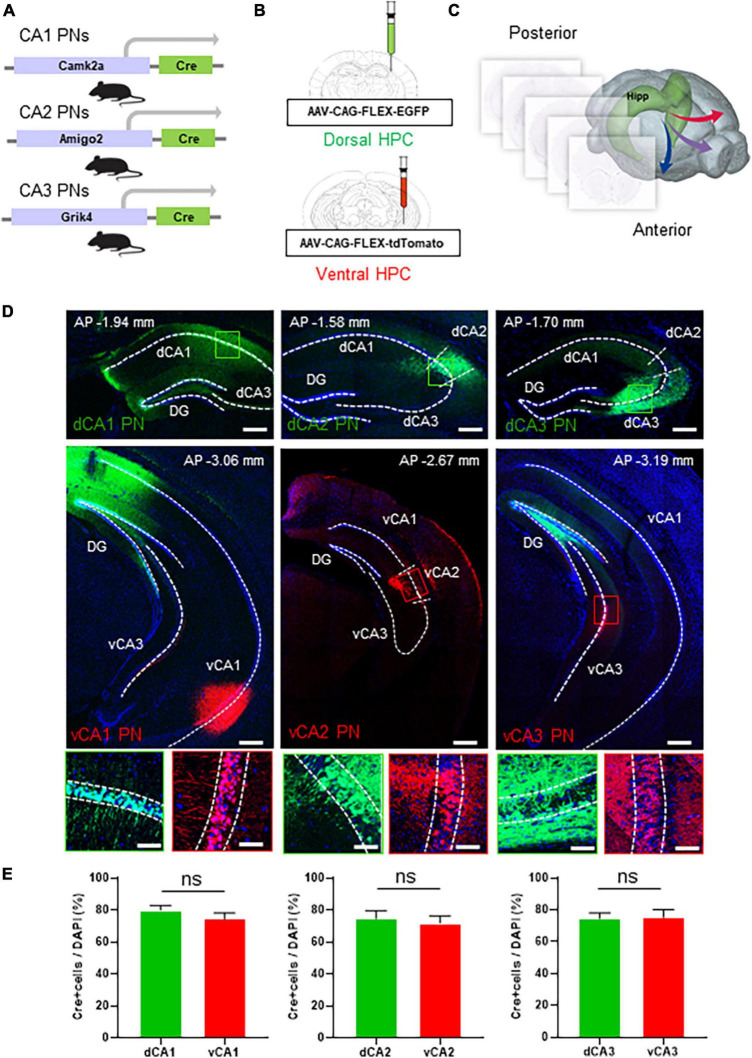
Dual colored labeling of each CA PNs type along the dorsoventral axis. Dual colored labeling of each CA PNs type along the dorsoventral axis. **(A)** Camk2a/Amigo2/Grik4-Cre transgenic mice **(B)** treated with packaged AAV strains and EGFP or tdTomato. An experimental design showing injection sites for the dorsoventral hippocampus tracing group. **(C)** Hippocampal projections are mapped in the whole brain region along the rostrocaudal axis. **(D)** Representative images of coronal brain sections containing the injection sites, and the magnifications of the PNs (the dorsal CA tracing group; the ventral CA tracing group). Scale bars = 200 μm; Scale bar: 50 μm in **(D)**. **(E)** Quantitative analysis of each CA PN specific Cre+ neurons coverages in pyramidal cell layer (two-tailed Student’s *t*-test or Mann–Whitney test, ns., no significant differenced, *N* = 5 in each group, female = 2, male = 3).

The hippocampus, a critical brain structure, comprises three representative subregions: CA1, CA2, and CA3. Notably, we excluded GCs from our investigation due to their role as a hippocampal subregion that does not contribute to extrahippocampal circuitry (Mei et al., 2017), thereby focusing our research on the remaining three subregions. To precisely delineate these subregions, we utilized cell type-specific Cre transgenic lines for CA1, CA2, and CA3 PNs, namely Camk2a-Cre, Amigo2-Cre, and Grik4-Cre (Tsien et al., 1996; Nakazawa et al., 2002; Hitti and Siegelbaum, 2014; Figure 1A). Each transgenic mouse line was subjected to stereotaxic injection of AAVs expressing fluorescent proteins in a Cre recombinase-dependent manner (*N* = 5 in each tracing group; male *n* = 3, female *n* = 2). By injecting EGFP- or tdTomato-expressing AAVs into the dorsal or ventral CA region, respectively, we could label Cre+ subpopulation of CA PNs with two distinct colors depending on its dorsoventral locations (Figure 1B and Supplementary Figure 1). This strategy enabled the discrimination of axon projections originating from the dorsal and ventral subpopulations of each CA PNs in the same animal (Figure 1B). In a series of coronal sections along the anterior-to-posterior axis of the whole brain, we verified dual-colored, cell type-specific labeling of dorsal and ventral subpopulations in the CA area (Figures 1C, D and Supplementary Figure 3). In the Camk2a-Cre line for CA1 PNs, the EGFP signal is in a broad area encompassing both proximal and distal areas of dCA1, whereas the tdTomato signal distributes specifically in vCA1. Furthermore, we confirmed that Amigo2-Cre and Grik4-Cre lines were useful for restricting these fluorescent protein expressions to their cognate cell types, CA2 PNs and CA3 PNs, respectively (Figure 1D). Notably, high-magnification images confirm that green and red fluorescence-labeled cells are specifically located within the pyramidal cell layers, where excitatory PNs are abundant, but not in the molecular layers, which contain sparse GABAergic inhibitory neurons (Figure 1D). Each Cre driver shows more than 70% coverage in the pyramidal cell layer at the injection site of each CA region (dCA1 = 79.92 ± 2.9%, vCA1 = 74.31 ± 3.85%; dCA2 = 74.62 ± 4.97%, vCA2 = 74.31 ± 4.55%; dCA3 = 74.53 ± 3.47%, vCA3 = 75.03 ± 4.71%) (Figure 1E). Despite the high level of fluorescence labeling within the CA PN subpopulation, we also found that a relatively small but significant portion of Cre- CA PNs (less than 30%) remains unlabeled. Therefore, our current analysis represents the projection pattern of the Cre+ CA PN subpopulation, but not that of the Cre- subpopulation, if there is any difference. The Camk2a-Cre line is predominantly active in Cre+ CA1 PNs but also shows weak activity in the GCs of the DG (Dragatsis and Zeitlin, 2000). Consequently, our results display EGFP signals in GCs and their axonal mossy fibers. This off-target EGFP distribution in DG GCs is likely due to the widespread infection of AAVs injected into the CA1 area (Figure 1D, upper panel). Additionally, fluorescence derived from Cre+ vCA1 PNs was observed in the molecular layer of the vCA3. This proximity suggests that the ventral DG is also infected with tdTomato-expressing AAV, similar to the dorsal hippocampus. Thus, the tdTomato signals in vCA3 of CamK2a-Cre mice likely originate from GCs in the ventral DG. Given that GCs do not project outside the hippocampus (Mei et al., 2017), the extrahippocampal projection of dorsoventral CA1 PNs is likely valid to be analyzed in the Camk2a-Cre line.

Amigo2 gene expression in CA2 PNs is limited to the far ventral area of the hippocampus, unlike other CA2 marker genes such as PCP4 and RGS14 (Allen brain atlas, #71250310). Consistently, Amigo2-Cre exhibits Cre activity from the dorsal to the immediate ventral side, but not in the far ventral side. Here, we labeled vCA2 PNs at bregma −3.00 mm in Amigo2-Cre, and thus tdTomato-labeled vCA2 PNs are relatively close to the EGFP-labeled dCA2 PNs compared to equivalent analyses in CA1 and CA3. Fluorescence-labeled CA2 PNs overlap with the CA2 PN marker, PCP4, in both the dorsal and ventral CA2 areas (Supplementary Figure 2), suggesting the specificity of the Amigo2-Cre line in fluorescence labeling of CA2 PNs along the dorsoventral axis.

AAVs often undergo long-distance axonal transport and induce transduction of brain regions distal to the injection site. However, the extent of axonal transport and distal transduction varies widely among AAV serotypes and viral injection sites in the brain (Rothermel et al., 2013). In response to the concern about the retrograde effect, we carefully re-examined our experimental setup to determine if this phenomenon could have influenced our results. We used the AAV serotype 1 for both EGFP and tdTomato labeling in each CA area. Importantly, we could not observe any retrograde labeling of presynaptic neurons projecting to the viral injection sites within the hippocampus. For instance, AAV injection into the CA3 area of Grik4-Cre mice did not result in labeling of DG GCs or their mossy fiber axons (Figure 1D). Similarly, AAV injection into the CA1 area of Camk2a-Cre mice did not label CA3 PNs. These observations suggest that retrograde transport did not occur in our experimental conditions, thereby supporting the validity of our interpretations. Collectively, we confirmed dual-colored, cell type-specific labeling of dorsal and ventral Cre+ CA PNs in the hippocampus using an intersectional approach of Cre-dependent AAVs and cell type-specific Cre transgenic animals.

### 3.2 Extrahippocampal projections of each CA PN type to distinct limbic areas

**FIGURE 2:**
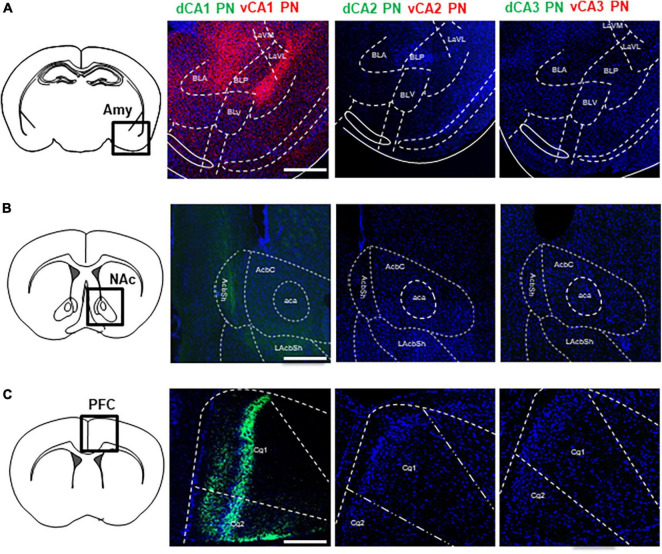
Extrahippocampal projections of CA PNs into the limbic areas. Extrahippocampal projections of CA PNs into the limbic area. **(A)** Each CA PNs fiber tracts patterns in BLA. **(B)** NAc **(C)** Cg. Scale bars = 200 μm in **(A–C)**. LaVM, lateral amygdala ventromedial part; LaVL, lateral amygdala ventrolateral part; BLA, basolateral amygdala; BLV, basolateral amygdala, ventral part; BLP, basolateral amygdala, posterior part; AcbC, nucleus accumbens core; AcbSh, nucleus accumbens shell; LAcbSh, lateral nucleus accumbens shell; Cg1, cingulate cortex 1; Cg2, cingulate cortex 2.

Firstly, we profiled the spatial distribution of axonal fibers from Cre+ CA PNs, with a particular focus on the limbic areas including the Amy, NAc, PFC, and LS. While all Cre+ CA PNs make common projections to the LS to be illustrated in the following figures, only CA1 PNs project their axonal fibers to the other limbic areas unilaterally (Figure 2). CA1 PNs make dense ipsilateral projections to Amy and PFC and much less to NAc. In contrast, the fluorescence-labeled axonal fiber of CA2 and CA3 PNs were not detectable in these limbic areas even with high-resolution microscopy. Furthermore, we could observe the clear difference between dorsal and ventral subpopulations of CA1 PNs in their axonal projections to these limbic areas. The EGFP-labeled axons from dCA1 PNs innervate into both NAc and PFC, but tdTomato-labeled axons from vCA1 PNs distribute only in Amy. Axonal fibers of dCA1 PNs are detected in the only shell region of the NAc (Figure 2B) and also in the layers 2/3 and 5 of the PFC (Figure 2C). In contrast, vCA1 PNs are detected in basolateral and lateral, but not in basomedial subfields of Amy (Figure 2A). Collectively, we demonstrated that Cre+ CA PNs, depending on their dorsoventral location in the hippocampus, exhibit distinct extrahippocampal connectivity with multiple limbic areas including Amy, NAc, and PFC.

### 3.3 Unilateral projections from CA1 PNs to the LS subfields

**FIGURE 3:**
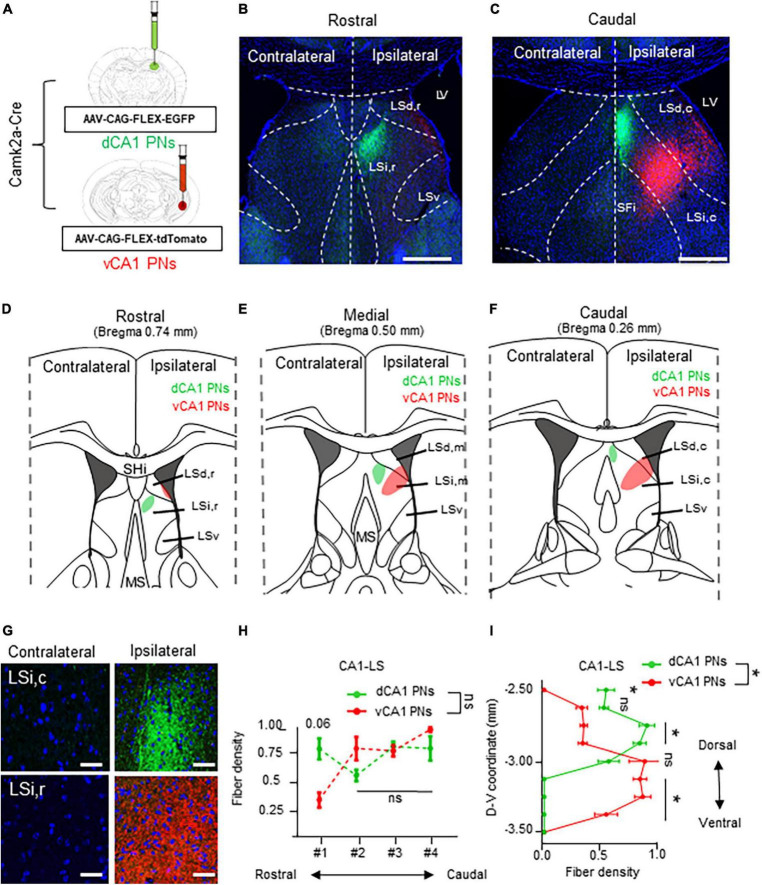
Unilateral projections of CA1 PNs into the LS and its 3-dimensional gradient. Unilateral projections of CA1 PNs into the LS and its 3-dimensional gradient. **(A)** Camklla-Cre transgenic mice treated with recombinant AAV strains in dorsal and ventral CA1. **(B,C)** Axonal fiber density of LS area along rostrocaudal axis. Scale bars = 200 μm in **(B,C)**. **(D–F)** Schematic image of dotted summary in LS subarea. dorsal and ventral PNs show distinct patterns in LS. **(G)** Higher magnification image of axonal fiber in LS. **(H,I)** Quantitative data of CA1 PNs fiber distributions along rostrocaudal and dorsoventral axis of the LS subfields. Scale bars = 50 μm in **(G)** (two-tailed Student’s *t*-test or Mann–Whitney test, ns., no significant differenced; *P < 0.05). LSd,r: dorsal LS, rostral part; LSi,r: intermediate LS, rostral part; LSd,m: dorsal LS, medial part; LSi,m: intermediate LS, medial part LSd,c: dorsal LS, caudal part; LSi,c: intermediate LS, caudal part; LSv: ventral part of LS; SHi: septohippocampal nucleus.

Next, we directly compared the projection pattern into the LS subfields from each Cre+ CA PNs. We explored the Cre+ CA PNs projection patterns within the 3-dimensional structure of the LS. We carefully partitioned the LS into discrete subdivisions along its rostrocaudal and dorsoventral axis. Each dCA1 and vCA1 PN is labeled with EGFP and the tdTomato and their axonal fiber in the LS or visualized in the series of coronal sections (Figure 3A). The dCA1 and vCA1 PNs exhibit a distinct ipsilateral projection to the LS, abstaining from projecting to the contralateral hemisphere (Figures 3B, C, G). Our observations highlighted innervation of the dorsal and ventral subpopulation of CA1 PNs in the 3-dimensional subfields of LS (Figures 3D–F). Specifically, dCA1 PNs preferentially project to the intermediate (LSi) and rostral parts (LSi,r) of LS in the rostral section (Figure 3D) and intermediate and caudal part (LSi,c) of LS in the caudal section (Figure 3F), whereas vCA1 PNs target the dorsal (LSd) and rostral parts (LSd,r) of the LS in the rostral section (Figure 3D) and LSi,c and dorsal and caudal part (LSd,c) of the LS in the caudal sections (Figure 3F). Notably, vCA1 PNs innervate to a broader area encompassing LSd and LSi regions, while dCA1 PNs exhibit projection to a highly confined region within LSi. dCA1 and vCA1 PNs exhibit equivalent densities of axonal fibers in the rostral section of LS (Figures 3D, H). However, vCA1 PNs, but not dCA1 PNs display the gradual increase in axonal density toward the caudal side of the LS (Figures 3F, H). dCA1 PNs innervate their fibers into the LS with the equivalent density of vCA1 PNs fibers, but vCA1 PNs exhibit gradually increase fiber distribution in the medial and caudal side, whereas dCA1 PNs fibers display identical density along the rostrocaudal axis (Figures 3E, F). Therefore, we found that dorsoventral CA1 PNs exhibit a gradient in their axonal density along the rostrocaudal axis of the LS, with significant differentiation of fiber density on the caudal side (Figure 3H). Furthermore, we assessed spatial distribution of CA1 PNs projections along the dorsoventral axis of the LS (Figures 3D–F, I). While dCA1 PNs primarily innervate the upper part of LS and exhibit a gradual decrease in fiber density toward the lower part, vCA1 PNs display opposite pattern in their axonal gradient along the dorsoventral axis of the LS. Therefore, dCA1 and vCA1 PNs exhibit different projection dominance along the dorsoventral axis of the LS (Figure 3I). These findings indicate a 3-dimensional gradient of extrahippocampal projections from the dorsoventral CA1 PNs to the selective subfields of LS.

### 3.4 Rostrocaudal gradient projections from CA2 PNs to the LS subfields

**FIGURE 4:**
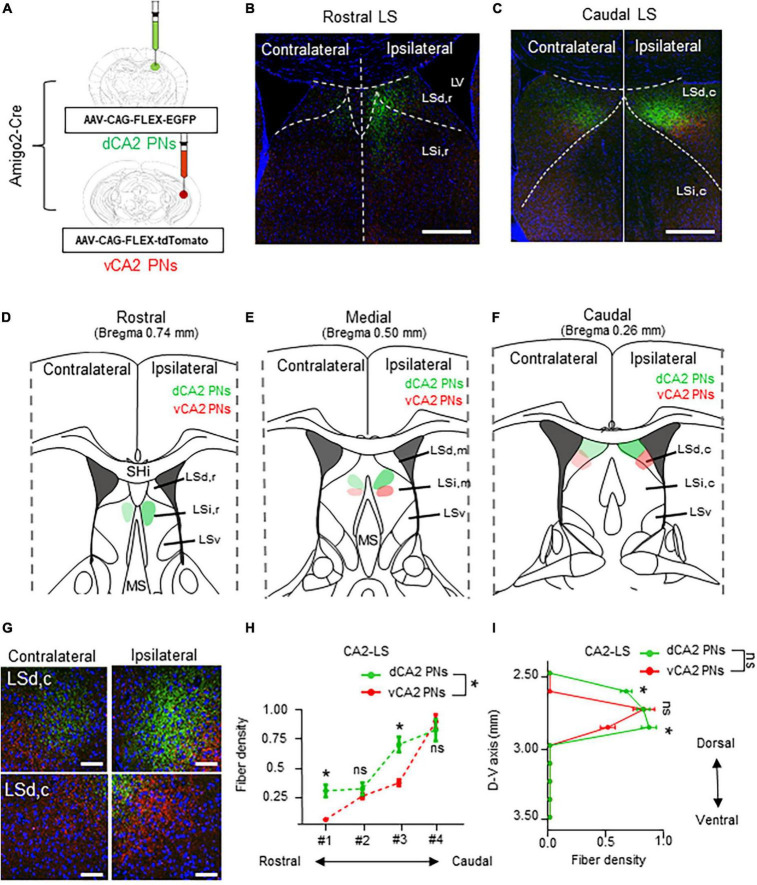
Lateralized bilateral projections of CA2 PNs into the LS and its rostrocaudal gradient. Lateralized bilateral projections of CA2 PNs into the LS and its rostrocaudal gradient. **(A)** Amigo2-Cre transgenic mice treated with recombinant AAV strains in dorsoventral CA2. **(B,C)** LS subregion receives inputs from hippocampus PNs along the dorsoventral and rostrocaudal axis. Scale bars = 200 μm in **(B,C)**. **(D–F)** Schematic image of serial sections of LS subregion and hippocampal fiber distributions. Along the rostrocaudal axis, dorsal and ventral PNs show distinct patterns in LS. **(G)** Higher magnification image of axonal fiber in LS. Scale bars = 50 μm in **(G)**. **(H,I)**: Quantitative data of CA2 PNs fiber distributions along rostrocaudal and dorsoventral axis of the LS subfields. dCA2 PNs and vCA2 PNs have displayed distinct patterns in LS along the dorsoventral axis, (two-tailed Student’s *t*-test or Mann–Whitney test, ns., no significant differenced; *P < 0.05).

Next, dorsoventral CA2 PNs are labeled their axonal fiber in the LS subfields with EGFP and the tdTomato each their dorsoventral axis (Figure 4A). The dCA2 and vCA2 PNs display a lateralized bilateral projection to the LS, unlike CA1 PNs. CA2 PNs innervate dense fibers to the ipsilateral LS and also slightly innervate the contralateral side of the LS (Figure 4G). Unlike CA1 PNs, axonal fibers from CA2 PNs exhibit a continuous gradient in LS along their dorsoventral location. Additionally, while dCA2 PNs cover a broader area than dCA1 fibers, vCA2 PNs display a narrower fiber pattern compared to vCA1 PNs (Figures 3B, C, 4B, C). Specifically, all CA2 PNs preferentially project to the LSi,r, of LS in the rostral section (Figure 4D) and LSd,c of LS in the caudal section (Figure 4F). Interestingly, dCA2 PNs display a broader and brighter fiber density than vCA2 PNs projection (Figures 4D–F). dCA2 PNs slightly innervate fibers to the LS, but axonal fibers from vCA2 PNs are not detected in the rostral section of LS (Figure 4D). Additionally, dorsoventral CA2 PNs display gradually increased fiber density toward the medial sections of LS (Figure 4E). Moreover, dCA2 PNs exhibit significantly higher density of axonal fibers in the caudal sections of LS (Figure 4E). Therefore, dorsal and ventral populations of CA2 PNs exhibit a gradient fiber pattern along the rostrocaudal axis of LS subfields, with significant differentiation of fiber patterns on the rostral and caudal sections of the LS (Figures 4D–F, H). Furthermore, we assessed the CA2 PNs projection along the dorsoventral axis of LS. Both dCA2 and vCA2 PNs specifically innervate to the upper part of the LS subfield without any signals on the lower part of LS. Unlike CA1, CA2 PNs project to similar positions in the LS along their dorsoventral location. Therefore, dCA2 and vCA2 PNs exhibit similar projection without fiber dominance along the dorsoventral axis of the LS (Figure 4I). Thus, CA2 PNs exhibit a lateralized bilateral projection to the LS, with a fiber gradient along the rostrocaudal axis of the LS depending on their dorsoventral location in the hippocampus.

### 3.5 Dorsoventral gradient projections from CA3 PNs to the LS subfields

**FIGURE 5:**
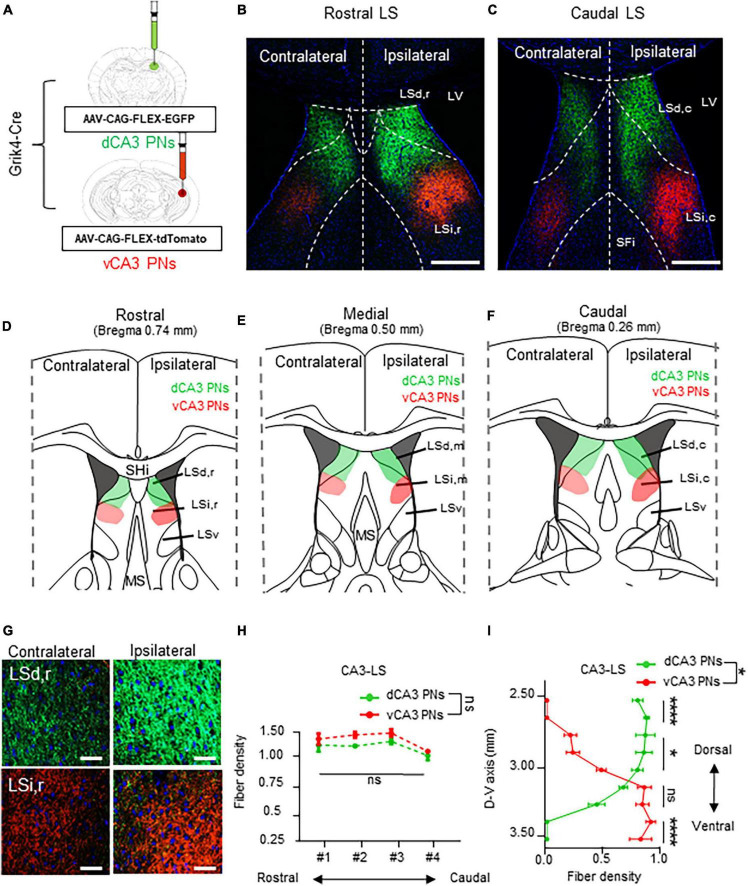
Bilateral projections of CA3 PNs into the LS and its dorsoventral gradient. Bilateral projections of CA3 PNs into the LS and its dorsoventral gradient. **(A)** Grik4-Cre transgenic mice treated with recombinant AAV strains in dorsoventral CA3. **(B,C)** LS subregion receives inputs from hippocampus PNs along the dorsoventral and rostrocaudal axis. Scale bars = 200 μm in **(B,C)**. **(D–F)** Schematic image of serial sections of LS subregion and hippocampal fiber distributions. Along the rostrocaudal axis of the LS, dCA3 PNs and vCA3 PNs show distinct patterns in LS subfields. **(G)** Higher magnification image of axonal fiber in LS. Scale bars = 50 μm in **(G)**. **(H)** The fibers from dCA3 and vCA3 PNs distribution patterns along rostrocaudal axis of the LS subfields (Mann–Whitney test, ns., no significant differenced). **(I)** Density of fibers from dCA3 and vCA3 PNs along the dorsoventral axis of the LS subfields (Mann–Whitney test, ns., no significant differenced; *P < 0.05; ****P < 0.001).

Finally, dorsoventral CA3 PNs label their axonal fibers in the LS subfields with EGFP and tdTomato along each dorsoventral axis (Figure 5A). The dCA3 and vCA3 PNs display bilateral projections to the LS subfield (Figure 5G). CA3 PNs also exhibit a continuous fiber gradient based on dorsoventral location, with the densest and widest coverage patterns in the LS subfield among Cre+ CA PN, regardless of their dorsoventral axis (Figures 5B, C). Specifically, dCA3 PNs project across LSd and LSi along the entire rostrocaudal axis of the LS. In contrast, the majority of vCA3 PNs project to the sole LSi region irrelevant to their rostrocaudal location within the LS (Figure 5D–F), while only small population projects to the edge of LSd,c of LS in caudal section (Figure 5F). Unlike other Cre+ CA PNs, each dCA3 and vCA3 PNs exhibit equivalent fiber density depending on the rostrocaudal axis of LS (Figure 5H). Furthermore, we profiled the CA3 PNs projection along the dorsoventral axis of LS. dCA3 PNs fibers mainly dominate the upper part of the LS and exhibit gradually diminishing fiber density toward the lower part of LS, whereas vCA3 PNs display an opposite pattern in their axonal gradient along the dorsoventral axis of the LS. Therefore, dCA3 and vCA3 PNs exhibit reverse dominance in projection fibers along the dorsoventral axis of the LS (Figure 5I). This intriguing finding suggests that CA3 PNs make unique projection patterns, with bilateral projections and dorsoventral gradients projected into selective subfields of LS.

## 4 Discussion

**FIGURE 6:**
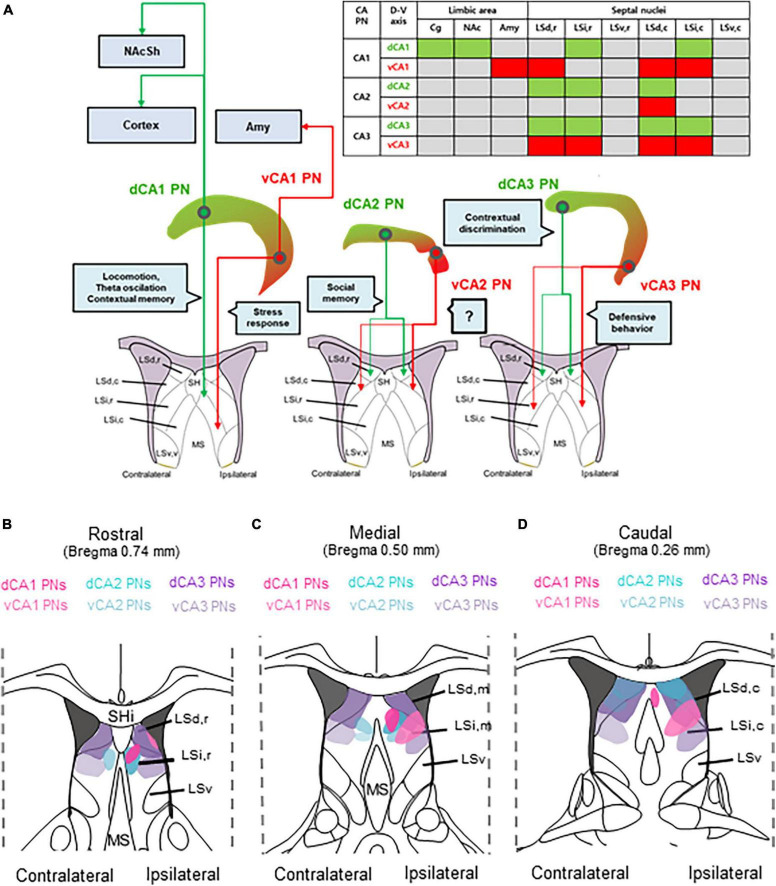
Comparative topography of extrahippocampal projections from CA PN types to the LS. Comparative topography of extrahippocampal projections from CA PN types to the LS. **(A)** Graphical summary of heterogeneity of CA PNs -septal circuit and each related function. **(B–D)** Serial schematic images of axonal fiber density in **(B)** rostral, **(C)** medial, and **(D)** caudal section of the LS subfields.

In the hippocampus, CA PNs make direct connections with multiple limbic areas through their extrahippocampal projections, critically influencing cognitive and affective behaviors. Here, we conducted a comparative analysis of the topological projection patterns of all three CA PNs using cell type-specific Cre transgenic lines for each CA PN type (Figure 1). Unlike CA2- and CA3 PNs projecting only to the LS, CA1 PNs exhibit a wider projection pattern to additional limbic areas including the Amy, NAc, and PFC (Figure 2). There is a dorsoventral difference in that dorsal CA1 PNs send their axonal fibers to NAc and PFC, ventral CA1 PNs project to Amy. Interestingly, all types of CA PNs, regardless of their dorsoventral locations, make axonal projections commonly to the LS, suggesting the recurrent extrahippocampal projection to the LS. Despite this conserved connectivity, these innervation patterns within LS subfields vary by CA PN type and their hippocampal positioning. Here, we show how these distinct patterns emerge in three-dimensional space along the rostrocaudal and dorsoventral axis of the LS (Figures 3–5). This topographical mapping significantly provides a framework for a better understanding of functional distinctions of CA PN types across the longitudinal axis of the hippocampus (Figure 6A).

To accurately map the projection patterns of dorsoventral CA PNs to various limbic areas, our study leveraged the precision of cell type-specific Cre transgenic animals combined with the targeted delivery of AAVs expressing fluorescent proteins in a Cre-dependent manner. This methodology employed transgenic mouse lines, including Camk2a-Cre for CA1 PNs, Amigo2-Cre for CA2 PNs, and Grik4-Cre for CA3 PNs, and validated to ensure gene delivery exclusively to each intended cell type (Tsien et al., 1996; Nakazawa et al., 2002; Hitti and Siegelbaum, 2014). This approach marks a significant advancement over traditional methods, which predominantly utilized antero- or retrograde-tracing dyes or Cre-independent AAVs. Such classical techniques often fall short in specificity, leading to the visualization of mixed projection patterns emanating from multiple neuronal types within the same area (Dong et al., 2009; Bienkowski et al., 2018). Critically, our methodology distinguishes itself by overcoming the limitations of non-specific labeling, which may render the accurate isolation of CA PN projections. Classical approaches using an anterograde tracing dye have constructed extrahippocampal projection maps in various limbic areas including the PFC, Amy, NAc, and, septum (Gaykema et al., 1991; Cenquizca and Swanson, 2007; Bienkowski et al., 2018). Similar to the previous studies, we observed that dCA1 PNs project to the NAc and PFC, and vCA1 PNs only project to the Amy (Figure 2). However, we demonstrated that CA PNs rarely make direct projections to the medial septum (MS) (Figures 3, 5) which is in sharp contrast to the findings from classical approaches. These discrepancies are attributable to the non-selective labeling of neuronal cell types in the CA subarea. In fact, GABAergic neurons in CA1 or CA3 make direct projection patterns to the MS, but not to the LS among multiple subfields of the septum (Jinno et al., 2007; Chiayu et al., 2022). Our study refined extrahippocampal projection patterns of each CA subfield while ensuring cell type-specificity. The specificity afforded by our use of CA PN-specific Cre lines enabled us to selectively label and observe the unique projection patterns of CA PNs without interference from interneuron projections. This precision in labeling is a pivotal improvement, eliminating the “noise” traditionally associated with non-specific approaches and revealing clear, cell-type-specific projection patterns. This precise approach not only provides a clearer understanding of hippocampal connectivity but also sets a new standard for specificity and accuracy in neural circuit mapping. The use of cell type-specific Cre lines and Cre-dependent AAVs in our study successfully highlights the precise projection patterns of CA PNs, extending beyond the intrahippocampal trisynaptic circuit, to selective limbic areas.

There is mounting evidence that the classical CA1-3 PNs all exhibit prominent within-cell-type heterogeneity (Cembrowski and Spruston, 2019). We also observed that CA PN type-specific Cre drivers can label over 75% of total PNs but not the entire population in a single transgenic line, likely due to the substantial heterogeneity of Cre expression within each CA PNs. Given this heterogeneity, Cre-driven labeling is restricted to the Cre+ subpopulation, providing only a partial representation of the topological projection of the target CA PNs. A recent study using sparse neuronal labeling combined with fluorescence micro-optical sectioning tomography (fMOST) techniques visualized the brain-wide axon collaterals of single neurons in the hippocampal subregions (Qiu et al., 2024). Interestingly, their results show that a single CA1 PN makes a more dominant projection to the MS than to the LS. This differs from our present findings, where Cre+ CA1 PNs project their axons predominantly to the LS and sparsely to the MS (Figure 3). This apparent discrepancy might be attributable to a putative difference between Cre+ CA1 PNs and the rest of the Cre- subpopulation, especially in their extrahippocampal projections. Future studies using differential labeling of these two distinct subpopulations within each CA PNs are warranted to examine such a possibility.

Here, we visualized the extrahippocampal projection of all three CA PNs and directly compared their topological patterns in parallel. We successfully visualized the differential projection of CA PN types into limbic areas. CA1 PNs project to multiple limbic areas including NAc, PFC, Amy, and LS, differently from CA2- and CA3 PNs projecting only to the LS. Furthermore, CA PNs exhibit notable differences in the degree of laterality in their axonal projections. CA1 PNs make unilateral projections to the target areas on the ipsilateral side (Figure 3), whereas CA3 PNs do bilateral projections almost equally to both hemispheres (Figure 5). Interestingly, CA2 PNs also send bilateral projections, but with strong dominance at the ipsilateral side (Figure 4). Furthermore, we delineated the distinct dorsoventral disparity of each Cre+ subpopulation of CA PNs in extrahippocampal projections into multiple limbic areas (Figure 6). While dCA1 PNs primarily projected their axonal fibers to the NAc, PFC, and LSi, vCA1 PNs exhibited a different pattern, projecting mainly to the Amy and LSd (Figures 2, 3). Likewise, dorsal and ventral subpopulations of CA2 and CA3 PNs displayed distinct projection patterns within the LS subfields (Figures 3, 4). Here we conducted differential fluorescence labeling of each Cre+ subpopulation of CA PNs only at two representative sites for the dorsal and ventral subpopulations, respectively. Thus, our present approach provides evidence to support the dorsal-ventral difference in CA PN axon projections but is yet to determine whether such difference exist in linear or non-linear gradients along the dorsoventral axis. Nonetheless, this unique extrahippocampal connectivity is likely associated with distinct functions of each CA PN even along the dorsoventral axis. dCA1 is implicated in reward-related memory processing via the PFC, NAc, and VTA. Plus, dCA1 also mainly facilitates memory processes via the LSi (Stuber et al., 2015; Trouche et al., 2019; Opalka and Wang, 2020; Liu et al., 2021; Terranova et al., 2023). Conversely, the vCA1 PNs-Amy and LS circuits regulate anxiety induction and emotional regulation (LeDoux, 2007; Root et al., 2015; Parfitt et al., 2017). Moreover, dCA2 plays a social memory and recognition through indirect input to the hypothalamus via the LSd (Wong et al., 2016; Leroy et al., 2018), whereas the function of vCA2 PN remains unknown (Besnard et al., 2019). Furthermore, dCA3 is associated with memory processes and encoding of reward-related contextual information through their indirect projections to the ventral tegmental area (VTA) or MS via the LSd (Jiang et al., 2016; Azevedo et al., 2020; Wirtshafter et al., 2021), whereas vCA3-LSi is associated with fear, anxiety, and reduced feeding (Besnard et al., 2020). Therefore, the unique projection patterns of each CA PN and its dorsoventral heterogeneity may provide important frameworks for functional differentiation.

The hippocampus receives cholinergic input from the MS and transmits its excitatory outputs to the LS which is associated with wide range of cognitive and affective behaviors (Risold and Swanson, 1997b; Sheehan et al., 2004). In this study, we constructed a fine map to illustrate the unique projection pattern of the hippocampus to the LS subregion (Figures 6B–D). This study elucidates how these distinct patterns exist in three-dimensional space along the rostrocaudal and dorsoventral axis of the LS. The LS is situated in the rostrodorsal portion of the septal region and is subdivided into LSd, LSi, and ventral LS (LSv) divisions (George and Franklin, 2013). According to the Allen Mouse Brain Atlas (Lein et al., 2007), the LS comprises caudal (LSc), rostral LS (LSr), and LSv regions. The LS is heterogeneous, composed of multiple subregions with molecular and functional distinctions. LSd is characterized by an enriched population of enkephalinergic neurons, projecting densely to the rostral hypothalamic medial zone (Risold and Swanson, 1997a; Sheehan et al., 2004). Conversely, LSi is notably rich in somatostatinergic neurons, projecting to the lateral hypothalamic and supramammillary nucleus (Sheehan et al., 2004; Deng et al., 2019). Here, we observed that LSd receives differential inputs from vCA1, dCA3, and both dCA2 and vCA2, whereas LSi receives bilateral input from both CA1 and vCA3 (Figures 3–5). Furthermore, each LS subregions are associated with highly distinct functions. LSd,c primarily facilitates memory processes with CA1 inputs. Whereas LSd,r is associated with spatial memory processes as well as social memory and recognition (Hunsaker et al., 2009; Besnard et al., 2019; Opalka and Wang, 2020). Here, we showed LSd receives different inputs from each CA PN type along its rostrocaudal axis (Figures 3–5). On the other hand, LSi activation is associated with fear and anxiety, and also with reduced feeding (Parfitt et al., 2017; Besnard et al., 2020). Available evidence suggests that the LSi and LSd exhibit opposing functions in promoting and suppressing learned fear via different CA3 inputs, respectively (Hunsaker et al., 2009; Besnard et al., 2019; Opalka and Wang, 2020). Interestingly, our mapping reveals that LSi receives inputs from both vCA1 and vCA3 PNs. In addition, all LS neurons send long-range projections to subcortical areas (Phelan et al., 1989). Indirect stimulation of LSd neurons inhibits VTA GABAergic neurons, leading to the disinhibition of VTA dopaminergic neurons. Furthermore, Lesions or chemogenetic inhibition in this circuit prevent context-induced but not cue-induced reinstatement of cocaine seeking (Jiang et al., 2016; McGlinchey and Aston-Jones, 2017). Additionally, slow gamma oscillations from the PFC are relayed through LSd neurons to the lateral hypothalamic area (LHA), increasing the firing rate of LHA neurons during food seeking. Optogenetic studies show this circuit is crucial for food seeking but not consumption (Carus-Cadavieco et al., 2017). LSd also promotes male aggression by activating the ventromedial hypothalamus (VMH) and inhibiting LSi neurons that project to VMH (Lin et al., 2011; Leroy et al., 2018). Moreover, LSi projections to the preoptic area and anterior hypothalamus mediate stress-induced behaviors (Anthony et al., 2014; Shin et al., 2018), respectively. Therefore, LS subfields make selective input and output regions, contributing to their functional heterogeneity. Thus, The Cre+ subpopulation of CA PNs, contingent on dorsoventral locations, are linked to the selective subfields of the LS through distinct axonal projections, thereby mediating functional cooperation between the hippocampus and the LS for cognitive and affection behaviors.

### 4.1 Limitations of the study

The hippocampus has a longitudinal structure extending along the dorsoventral axis. Here, we conducted differential fluorescence labeling of each CA PN type at two representative sites for the dorsal and ventral subpopulations, respectively. Due to the technical challenges of analyzing more than two fluorescent proteins in an animal, we could not expand labeling sites to cover the entire hippocampus (Figure 1D). Elaborate tracing systems compatible with multisite and multicolor labeling are required to determine whether CA PN projections exhibit dorsoventral differences in linear or non-linear gradients. Here we utilized CA PN-specific Cre transgenic animals in this study. However, it remains unclear whether the Cre drivers used here represent the entire population of each CA PN (Figure 1E). With growing evidence about single-cell heterogeneity even within a neuronal type, we cannot rule out that Cre- subpopulation may exhibit different projection pattern as compared to Cre+ one of each CA PN type that we visualized here. For dual fluorescence labeling of dorsal and ventral subpopulations of all three Cre+ CA PNs, we utilized tdTomato and EGFP throughout the present study. However, further studies using cross-labeling approaches are required to address whether the topological pattern of extrahippocampal projection is influenced by intrinsic differences between the two fluorescent proteins. There is growing evidence indicating sex differences in neurodevelopment and circuitry (Cindy et al., 2013; Shinji et al., 2014). Although the present study did not observe any notable sex differences, especially in the extrahippocampal projection patterns of Cre+ CA PNs, future studies with larger cohorts are warranted for systematic comparisons to examine this possibility carefully.

## 5 Conclusion

In our study, we map the extrahippocampal projection patterns of Cre+ CA PNs beyond their trisynaptic circuit through an intersectional approach using cell-type-specific Cre animals and fluorescence protein AAVs. We visualize the direct connectivity of Cre+ subpopulation of CA PNs with various limbic areas. Our findings indicate that while Cre+ CA1 PNs project widely to limbic areas including the Amy, NAc, and PFC, Cre+ CA2 and CA3 PNs primarily project to the LS. Notably, despite cell type differences, all Cre+ CA PNs project to the LS, suggesting its significant association within the limbic circuitry. Notably, the Cre+ CA PNs project into a highly selective LS subfield depending on CA neuronal types and their dorsoventral locations, which may underlie their functional differentiation in the hippocampus. This detailed topographical mapping provides the neuroanatomical basis of the underlying functional distinctions among CA PN types.

## Data availability statement

The original contributions presented in this study are included in the article/Supplementary material, further inquiries can be directed to the corresponding author.

## Ethics statement

The animal study was approved by the Institutional Animal Care and Use Committee. The study was conducted in accordance with the local legislation and institutional requirements.

## Author contributions

JL: Writing – original draft, Writing – review & editing. JP: Writing – review & editing. MJ: Writing – review & editing. S-JO: Writing – review & editing. J-HY: Writing – review & editing. Y-SO: Writing – original draft, Writing – review & editing.

## Supplementary material

Supplementary Figure 1 Sections of the brain showing injection site locations. Viral injection/expression sites for experiments in Figures 1–5. Coronal brain sections at the bregma levels showing viral dorsal (green dots) and ventral injection sites (red dots)/expression areas (green and red circles with size change). Scales of circles represent expression width and brightness of circles represent labeled cell densities. (*N* = 5, F = female, M = male) **(A,B)** Camk2a-Cre mice (in Figures 1–3). **(C,D)** Amigo2-Cre mice (in Figures 1, 2, 4). **(E-F)** Grik4-Cre mice (in Figures 1, 2, 5).

Supplementary Figure 2 Genetic identify of the CA2 PNs using the Amigo2-Cre mouse line. Genetic targeting of the CA2 subfield using the Amigo2-Cre mouse line **(A,B)** Unilateral injections of Cre-dependent AAVs in Amigo2-Cre mice resulted in specific expression of **(A)** EGFP (dorsal) and **(B)** tdTomato (ventral) in CA2 PNs. **(C–H)** Magnified images of boxed area in **(A,B)**. **(C)** EGFP (green). **(D,G)** PCP4 staining (magenta). **(E)** Merge of **(C,D)** showing EGFP and PCP4 overlap with DAPI (blue). **(F)**, tdTomato staining (red). **(H)** merge of **(F,G)** showing tdTomato and PCP4 overlap with DAPI. **(I)** Quantification of the spatial distribution of dorsal and ventral CA2 PNs. The ratio for colocalization was determined by dividing the number of colocalized cells by the total number of viral labeled cells. Scale bars, 200 μm in **(A,B)**, 50 μm in **(C–H)**.

Supplementary Figure 3 Serial sections of each CA subpopulations. Representative images showing distributions of the Cre+ neurons located in the hippocampus. Scale bar: 300 μm.
