# Supplementary figures and images for: Anatomical topology of extrahippocampal projections from dorsoventral CA pyramidal neurons in mice
Source: Front Neuroanat. 2024 Jul 23;18:1421034. doi: 10.3389/fnana.2024.1421034 (PMC11300266; doi:10.3389/fnana.2024.1421034)

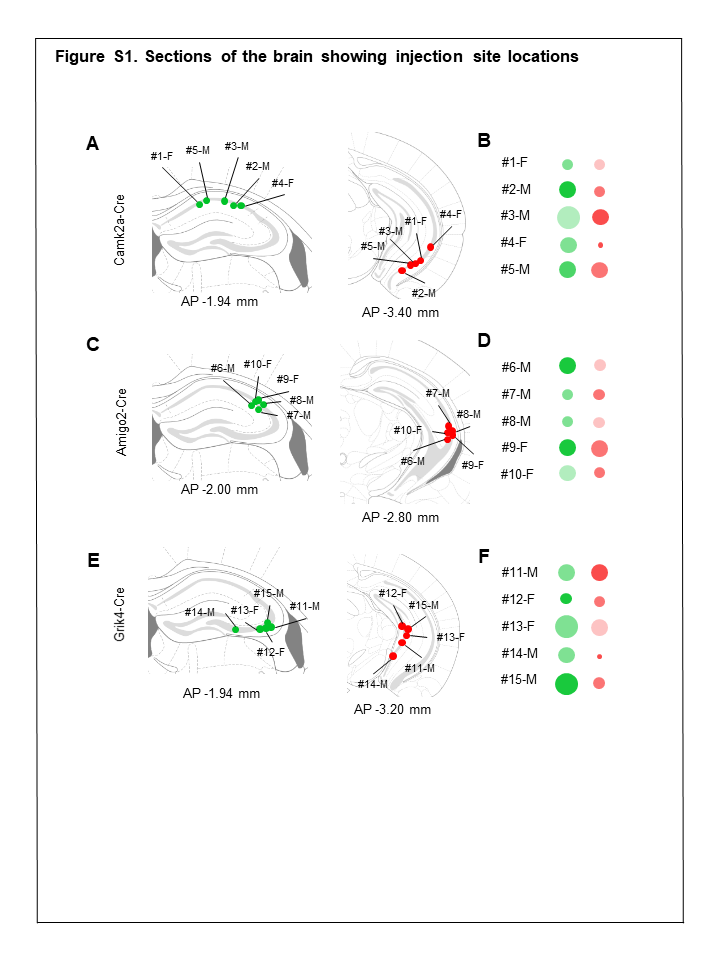

Supplement: Supplementary Figure 1 — Sections of the brain showing injection site locations. Viral injection/expression sites for experiments in Figures 1–5. Coronal brain sections at the bregma levels showing viral dorsal (green dots) and ventral injection sites (red dots)/expression areas (green and red circles with size change). Scales of circles represent expression width and brightness of circles represent labeled cell densities. (N = 5, F = female, M = male) (A,B) Camk2a-Cre mice (in Figures 1–3). (C,D) Amigo2-Cre mice (in Figures 1, 2, 4). (E-F) Grik4-Cre mice (in Figures 1, 2, 5). [file Image_1.TIF]

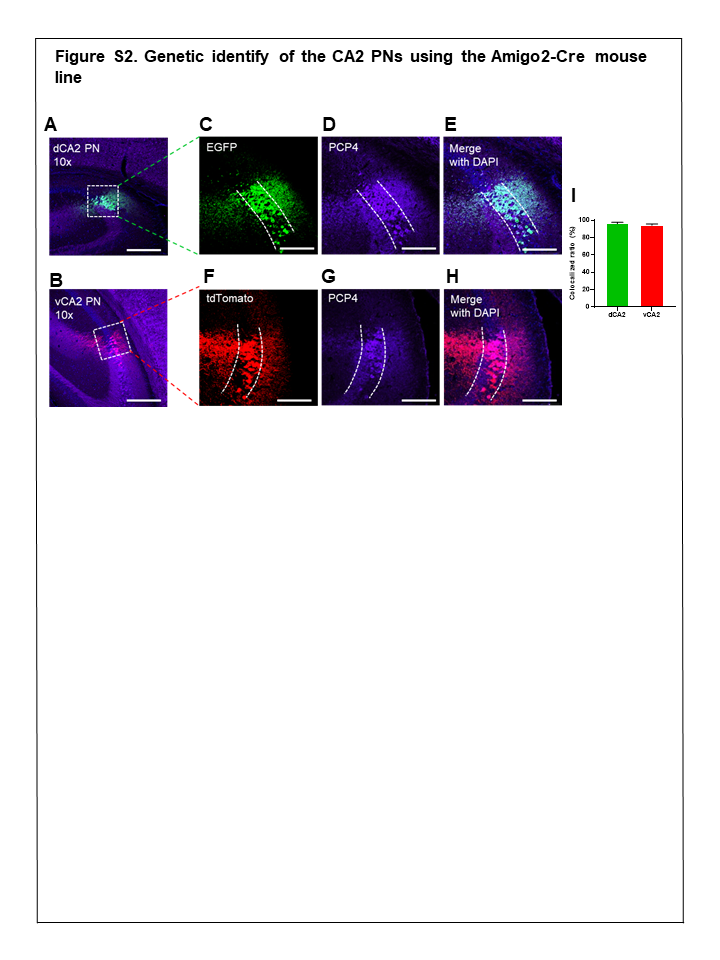

Supplement: Supplementary Figure 2 — Genetic identify of the CA2 PNs using the Amigo2-Cre mouse line. Genetic targeting of the CA2 subfield using the Amigo2-Cre mouse line (A,B) Unilateral injections of Cre-dependent AAVs in Amigo2-Cre mice resulted in specific expression of (A) EGFP (dorsal) and (B) tdTomato (ventral) in CA2 PNs. (C–H) Magnified images of boxed area in (A,B). (C) EGFP (green). (D,G) PCP4 staining (magenta). (E) Merge of (C,D) showing EGFP and PCP4 overlap with DAPI (blue). (F), tdTomato staining (red). (H) merge of (F,G) showing tdTomato and PCP4 overlap with DAPI. (I) Quantification of the spatial distribution of dorsal and ventral CA2 PNs. The ratio for colocalization was determined by dividing the number of colocalized cells by the total number of viral labeled cells. Scale bars, 200 μm in (A,B), 50 μm in (C–H). [file Image_2.TIF]

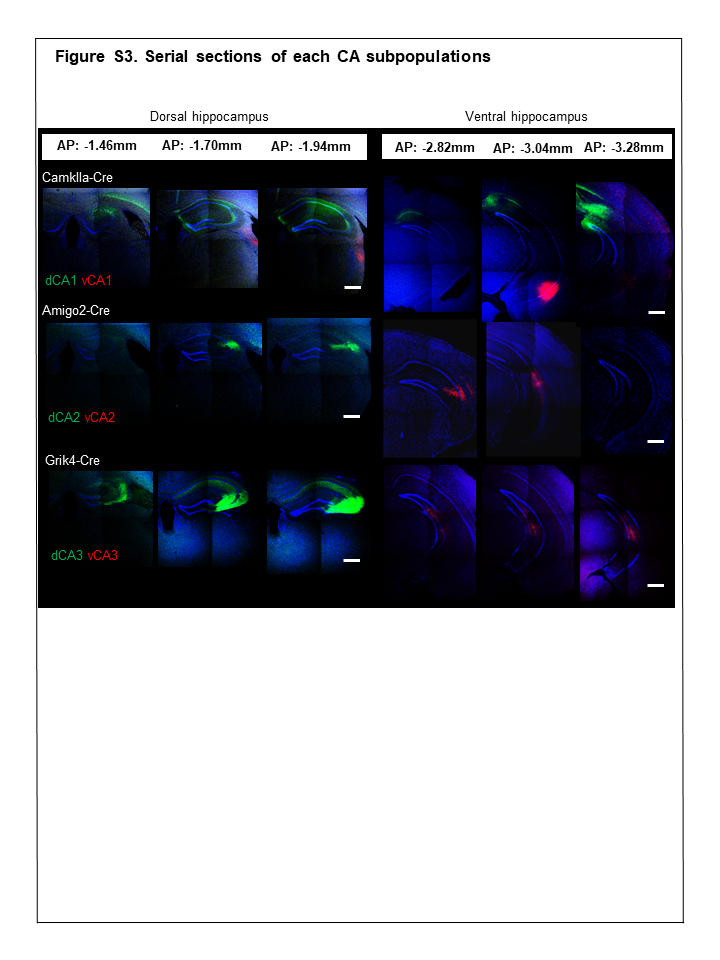

Supplement: Supplementary Figure 3 — Serial sections of each CA subpopulations. Representative images showing distributions of the Cre+ neurons located in the hippocampus. Scale bar: 300 μm. [file Image_3.tif]
